# Supplementary figures and images for: Prognostic value of FoxP3 and CTLA-4 expression in patients with oral squamous cell carcinoma
Source: PLoS One. 2020 Aug 12;15(8):e0237465. doi: 10.1371/journal.pone.0237465 (PMC7423125; doi:10.1371/journal.pone.0237465)

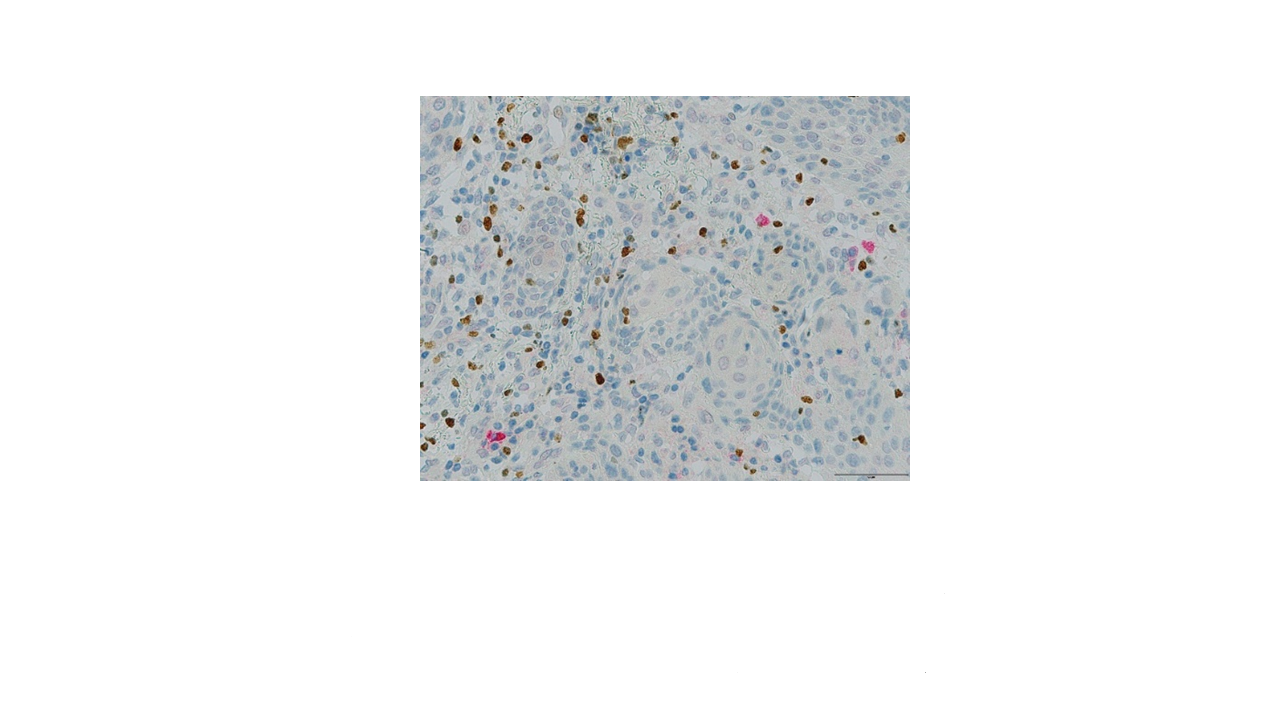

Supplement: S1 Fig — Brown and pink staining represent FoxP3+ T-cells and CTLA-4+ cells, respectively. CTLA-4+ cells were localized around FoxP3+ T-cells. (TIF) [file pone.0237465.s001.tif]
