# Supplementary material for: Prognostic value of FoxP3 and CTLA-4 expression in patients with oral squamous cell carcinoma
Source: PLoS One. 2020 Aug 12;15(8):e0237465. doi: 10.1371/journal.pone.0237465 (PMC7423125; doi:10.1371/journal.pone.0237465)
Supplement: Attachment — Submitted filename: Response_to_reviewer_s_comments_.docx [file pone.0237465.s002.docx]

**RESPONSE TO REVIEWER COMMENTS**

**Reviewer 1:** Koike et al. have used immunohistochemistry to evaluate the presence of tumor-infiltrating FoxP3⁺ T-cells and CTLA-4⁺ cells in 137 patients with oral squamous cell carcinoma (OSCC) regarding the prognostic value of tumor-infiltrating lymphocytes. Five-year overall survival, disease-specific survival, and recurrence-free survival were favorable in patients with high numbers of FoxP3⁺ T-cells. Recurrence-free survival and metastasis-free survival were decreased in patients with high numbers of CTLA-4⁺ cells. In conclusions, the presence of FoxP3⁺ T-cells in the parenchyma of the invasive front may be a useful prognostic factor. FoxP3⁺ T-cells may exert site-specific anti-tumor effects but may not play an immunosuppressive role in OSCC.
The claims are properly placed in the context of the previous literature. The experimental data support the claims. The manuscript is written clearly enough that most of it is understandable to non-specialists. The authors have provided adequate proof for their claims, without overselling them. The authors have treated the previous literature fairly. The paper offers enough details of methodology so that the experiments could be reproduced.

**Response:** Thank you for your kind comments.

**Reviewer 2:** It is interesting to show that FoxP3⁺ T-cells in the parenchyma is associated with OS. The key question is if this conclusion is robust. To test this, I suggest that authors take 60% of the samples to re-examine the association, with 100 times to see if the association is still OK. In addition, authors should discuss the germline variants associated with the NK cells are also linked to the OS (PMID: 31483464), which is related the report here.

**Response:** We have consulted a specialist in oral pathology and repeatedly verified these data to ensure that the association is reproducible. Thank you for recommending the study by Xu et al.; in the Introduction, we have added that germline variants associated with NK cells are also linked to clinical outcomes and cited the study (P4 L55-59).

**Reviewer 3:** Manuscript PONE-D-20-11094 describes the results from investigating the prognostic value of FoxP3+ CTLA-4+ cells density in oral squamous cell carcinoma patients. Using a respectably sized cohort of 137 subjects who underwent definitive surgery with no chemotherapy or radiotherapy prior to surgery and with available FFPE sections, the authors measured densities of FoxP3+ T-cells and CTLA-4+ cells in 4 different locations and determined their relationships with several survival parameters. The authors found that high density of FoxP3+ T-cells in the parenchyma of the invasive front was associated with favorable survival, while high density of CTLA-4+ cells in the same location were not favorable for survival. The authors also suggest several mechanisms which may underlie the relationship between cell densities and survival, which will require more detailed examinations to be elucidated.
Overall, this is a clearly written manuscript presenting new evidence for the usefulness of FoxP3+ T-cell and CTLA-4+ cell density as prognostic biomarkers in oral squamous cell carcinoma.
To improve this manuscript, this reviewer suggests addressing the following questions:
1. What is the relationship between the density of FoxP3+ T-cells and CTLA-4+ cells?
2. Are the associations between FoxP3+ T-cells and CTLA-4+ cells and survival independent of each other?

**Response 1:** We confirmed by double staining that FoxP3^+^ T-cells and CTLA-4^+^ cells were not colocalized, though CTLA-4^+^ cells were present around FoxP3^+^ T-cells (P27 L370-371). Therefore, the density of FoxP3^+^ T-cells and CTLA-4^+^ cells appeared to be unrelated.

**Response 2:** The associations between FoxP3^+^ T-cells and CTLA-4^+^ cells and survival were evaluated using a χ^2^ test. None of the associations reached statistical significance (IF stroma; p=1.00, IF parenchyma; p=0.13, TCe stroma; p=0.15, TCe parenchyma; p=0.89). Therefore, FoxP3^+^ T-cells and CTLA-4^+^ cells are independent prognostic factors, but mutual evaluation in a larger cohort is recommended to gain a better understanding of this relationship (P28 L382-385).
